# Investigation of how accurately individuals with hemiparetic stroke can mirror their forearm positions

**DOI:** 10.1371/journal.pone.0250868

**Published:** 2021-04-30

**Authors:** Netta Gurari, Justin M. Drogos, Julius P. A. Dewald

**Affiliations:** 1 Department of Physical Therapy and Human Movement Sciences, Northwestern University, Chicago, Illinois, United States of America; 2 Department of Mechanical Engineering, Northwestern University, Evanston, Illinois, United States of America; 3 Department of Biomedical Engineering, Northwestern University, Evanston, Illinois, United States of America; West Virginia University, UNITED STATES

## Abstract

Current literature suggests that greater than 50% of survivors of a stroke cannot accurately perceive where their upper extremity is positioned. Our recent work demonstrates that the extent to which this perception is affected can depend on how the task is performed. For example, individuals with stroke who have a deficit in mirroring the position of their passively-placed paretic forearm during a between-arms task may accurately reproduce the position of their actively-controlled paretic forearm during a single-arm task. Moreover, the ability of individuals with various types of unilateral lesions to locate their thumb can depend on whether they reach for their paretic thumb or non-paretic thumb. Consequently, we investigated to what extent the accuracy of individuals post-hemiparetic stroke in mirroring forearm positions on a between-arms task is influenced by various conditions. Eighteen participants with hemiparetic stroke rotated their reference forearm to a target position, and then rotated their opposite forearm to concurrently mirror the position of their reference forearm. This task was performed when participants referenced each forearm (paretic, non-paretic) at two target positions (extension, flexion) for two modes of limb control (passive, active). We quantified for every testing scenario of each participant their position-mirroring error. The number of times for which participants were classified as having a deficit was least when mirroring forearm positions at the flexed position when referencing their non-paretic forearm. Additionally, the difference in the magnitude of errors when participants referenced each arm was greater during active than passive movements. Findings from this study provide further evidence that the accuracy with which individuals post stroke perceive the position of their limbs can depend on how a task is performed. Factors to consider include whether movements are active versus passive, which limb is referenced, and where the limb is positioned.

## Introduction

Existing literature suggests that by the year 2030 more than four million survivors of a stroke in the United States will inaccurately perceive where their paretic limb is positioned and how it is moving [1–4]. This is largely based on clinical assessments that request individuals to perform tasks such as mirroring limb positions [5]. Deficits in mirroring limb positions during such assessments may have a negative impact on motor and functional abilities, increase length of hospital stay, and decrease quality of life [6]. However, our recent work [7, 8], as well as unpublished pilot testing (see S1 Video), indicates that generalizations drawn based on such assessments may be misleading. We demonstrated that depending on how a task is performed in an assessment, an individual post stroke may or may not be classified as having a deficit. Factors that could potentially lead to differing classifications include how the limb is controlled (e.g., passively imposed versus actively controlled movements) [9–23], which limb is used and how (e.g., paretic versus non-paretic) [24, 25], and the limb’s position (e.g., extended versus flexed) [12, 14, 26]. Hence, we indicate that a need exists to accurately, as well as comprehensively characterize one’s limb position perception deficits. In this way, a more complete understanding for the extent of the challenges one experiences in perceiving their limb(s) after a stroke can be obtained.

Current assessments that characterize deficits in perceiving where one’s limb is positioned and how it is moving typically require individuals with hemiparetic stroke to indicate what they feel when or after movements are imposed on their paretic limb [3, 4, 8, 27, 28]. Tasks include i) mirroring the position at which a paretic limb is placed using the non-paretic limb, ii) detecting when a movement that is imposed on the paretic limb occurs, and iii) detecting the direction of a movement that is imposed on the paretic limb. Such assessment methods were designed in part to account for the fact that individuals with hemiparetic stroke have motor impairments that can interfere with their limb control. Even so, these assessments provide limited insight since they do not indicate whether a deficit occurs when individuals with stroke actively control their paretic limb.

In our earlier work, we developed a new method that permits assessment of individuals post stroke when they actively move their paretic limb [7]. Our approach was designed to control for motor impairments so as to expose the perceptual deficit. We investigated how accurately individuals with hemiparetic stroke could reproduce the position at which they previously placed their paretic forearm and non-paretic forearm, independently. This approach was employed for a single-arm task, however, has not yet been applied to a between-arms task. As such, in this study we investigated whether individuals with hemiparetic stroke can accurately mirror their forearm positions between arms when controlling movements at both limbs.

Our understanding of how accurately individuals with hemiparetic stroke can mirror their forearms on a between-arms task remains unknown not only during active movements, but also during solely passive movements. The processes governing perception may differ in individuals without neurological impairments depending on whether movements are active or passive [9–23]. For example, during active control the alpha-gamma motor neuron coactivation may heighten muscle spindle sensitivity [29, 30]. Additionally, a copy of the motor command may provide additional information as to the limb’s position when the person generates their own movements [19, 31]. As such, we were motivated to determine whether accuracy in mirroring forearm positions differs if individuals with hemiparetic stroke control their movements (active) versus have their limbs controlled for them (passive).

Moreover, of particular interest is whether the arm referenced when mirroring forearm positions influences one’s accuracy. Hirayama et al. demonstrated that individuals with unilateral lesions who have a deficit when using their non-paretic hand to locate their passively-placed paretic thumb do not have a deficit when using their paretic hand to locate their passively-placed non-paretic thumb [24]. In addition, our unpublished pilot work revealed that the ability of an individual with clinically-assessed absent kinaesthesia to mirror forearm positions depended on whether the paretic versus non-paretic arm was referenced (see S1 Video). As such, we were inspired to determine whether the arm individuals with hemiparetic stroke reference during a between-arms task influences how accurately they can mirror forearm positions.

Arm position has also been shown to affect an individual’s accuracy in perceiving where their limb is positioned [12, 14, 26]. Therefore, in line with our earlier work, we were interested in determining how accurately participants could mirror their forearm position at two target positions—extension and flexion [14]. Also aligned with our previous work, we employed methods that included a custom robotic system and automated protocol [14]. In turn, we could ensure that the assessment methods were highly controlled and reproducible, and that the outcome measures were objective with a good resolution.

Below we present a human subject study in which we assessed the extent to which the arm referenced influenced how accurately eighteen individuals with hemiparetic stroke could mirror their forearm positions at two positions during purely active and purely passive movements.

## Materials and methods

The same procedures employed here were previously used for testing in individuals without neurological impairments (i.e., controls) [14]. As such, we adapted text from [14] to describe our procedures here.

### Participants

The Northwestern University Institutional Review Board approved this study (STU00021840). Each participant provided written informed consent and was monetarily compensated for their time. Co-author Dr. Justin Drogos, a licensed physical therapist, screened participants and assessed their sensorimotor abilities using the upper-extremity Fügl-Meyer Motor Assessment (UE FMA) [32] and revised Nottingham Sensory Assessment (rNSA). T1/T2 MRI scans and medical records were used to determine based on visual inspection the location(s) of lesion(s) in each participant’s brain. Participant requirements included: having a unilateral brain lesion greater than one year prior; not having diagnoses that result in sensory and motor deficits at the upper extremities beyond those resulting from the stroke; not using antispastic agents, e.g., baclofen, in the past six months; ability to detect the direction in which the paretic forearm was passively rotated during the rNSA elbow kinaesthetic sensation test (required for the passive mode of limb control) [4]; and ability to control forearm movements at <10°/s (required for the active mode of limb control).

We include in this manuscript data from nine individuals without neurological impairments (i.e., controls) who completed the same experimental trials as the participants with hemiparetic stroke [14]. As such, we could compare the results of our participants with stroke to similarly-aged controls. The mean (standard deviation; range) age for the controls was 59 (7; 46-69), with seven who were female and two who were male.

### Testing scenarios

The participant mirrored forearm positions at two target positions (extension, flexion) for two modes of limb control (passive, active) when referencing each arm (paretic, non-paretic). Initially, one of the forearms, which we denote as the reference forearm, rotates to the reference angle. Following, the opposite forearm, which we denote as the indicator forearm, rotates to mirror the position of the reference forearm. The angle at which the indicator forearm mirrors the reference forearm is identified as the indicator angle.

### Setup

**Fig 1:**
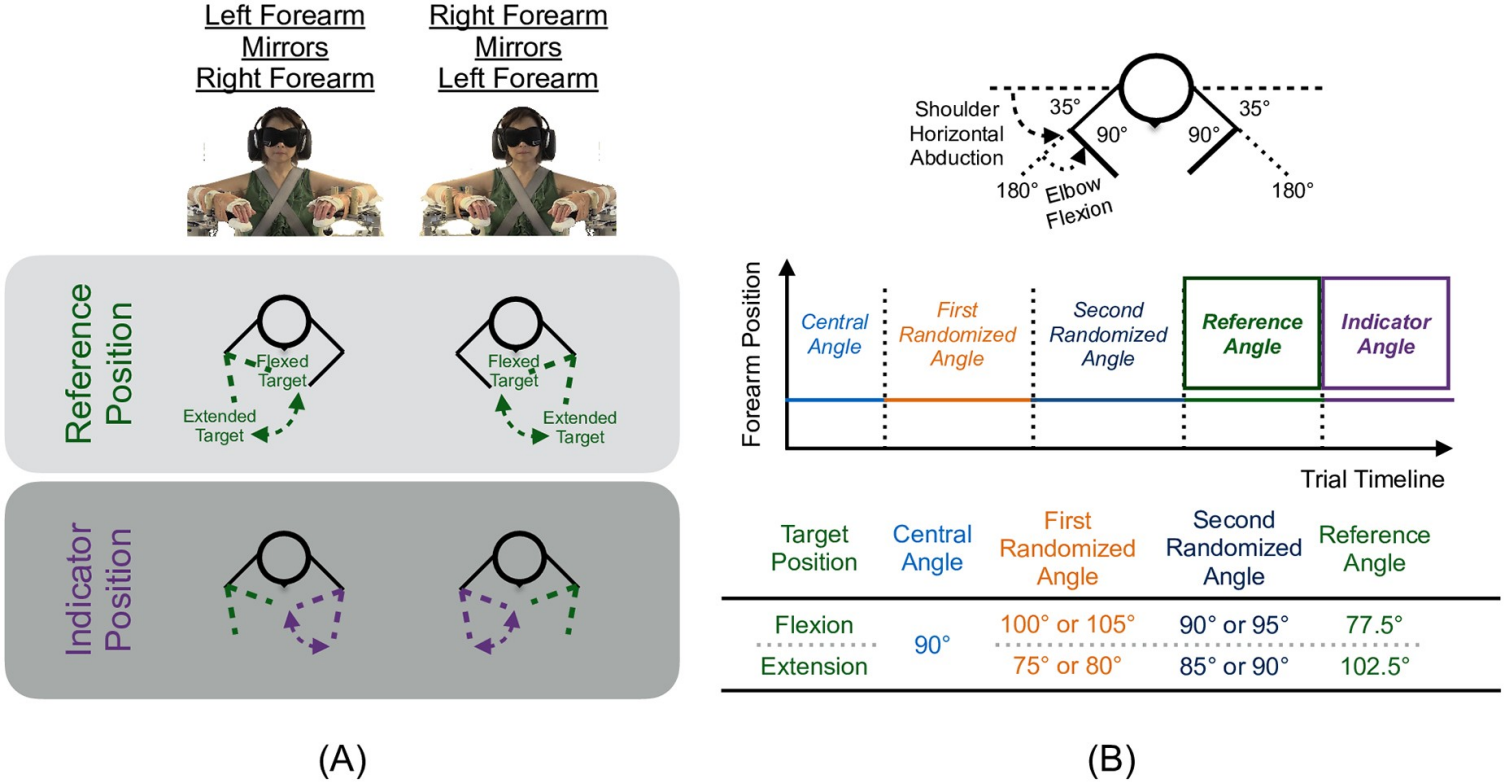
Experimental procedures. (A) The participant’s reference forearm, shown in green, rotated to the target position, and their indicator forearm, shown in purple, rotated to mirror their reference forearm’s position. This task was performed when the participant’s forearms were moved passively and actively to an extension and flexion target. (B) Top: Top-down visual depiction of how the shoulder elbow angles were defined, with the dashed horizontal line and error indicating 35° in shoulder horizontal abduction and the solid and dotted lines identifying 90° and 180°, respectively, in elbow flexion. Middle: Summary of the positions to which the participant’s forearms rotated throughout a trial. Bottom: Angles to which the participant’s reference forearm rotated during an extension and flexion trial. The individual in this manuscript has given written informed consent, as outlined in the PLOS consent form, to publish these case details. *This figure was adapted from Gurari et al., 2018 ©* [14].

The experimental setup is shown in Fig 1A. The participant was positioned in a Biodex chair (Shirley, NY, USA) with their upper extremities in anatomically mirrored positions of 85° shoulder abduction and 35° shoulder horizontal abduction from the frontal plane. Their forearms were casted and attached to a custom single-degree-of-freedom robotic device and custom single-degree-of-freedom measurement device. A US250 Encoder (Peabody, MA, USA) and US Digital MA3 Miniature Absolute Magnetic Shaft Encoder (Vancouver, WA, USA) identified the angular position of the custom robotic device and custom measurement device, respectively. A motor on the custom robotic device either controlled or permitted the participant to control their movements. An experimenter rotated, via a handle, the custom measurement device to a specified angular position at a desired speed when movements were passive, and the participant rotated the custom measurement device to a specified angular position at a desired speed when movements where active.

### Trial

The trial sequence for the passive and active trials is summarized in Fig 1B.

#### Passive trial

During the passive mode of limb control, the participant remained relaxed while their forearms were rotated for them. Their reference forearm was rotated by the custom robotic device, and their indicator forearm, which was fixed to the custom measurement device, was rotated by the experimenter. To begin, the participant’s forearms were stretched in extension and flexion at approximately 90°/s to avoid the effect of muscle thixotropy on position-mirroring ability [22, 33, 34]. Following, the forearms were rotated to the central angle (90°). Throughout the remaining movements, audio cues (‘In’, ‘Out’, ‘Stop’) played aloud indicating the actions occurring at the reference forearm and indicator forearm, respectively. As such, the audio cues throughout a passive trial were comparable to the audio cues throughout an active trial. The remaining forearm movements were restricted to <10°/s to ensure comparable interaction speeds and, in turn, torque responses from each device [14, 35]. The participant’s reference forearm rotated to the first randomized angle, followed by the second randomized angle. The inclusion of these randomized angles ensured that the participant could not mirror forearm positions based on timing cues. Next, the reference forearm rotated to the reference target position (extension: 102.5°, flexion: 77.5°) and held still for 4 s. Finally, the participant instructed the experimenter, using the commands ‘In’, ‘Out’, and ‘Stop’, on how to rotate their opposite indicator forearm to mirror the position of their reference forearm. The participant stated ‘match’ when their forearms were perceived as being in an identical mirrored position. This marked the end of the trial.

#### Active trial

During the active mode of limb control, the participant controlled the movements at their reference forearm and indicator forearm. To begin, the participant’s forearms were rotated between approximately 70° and 150° at approximately 90°/s to avoid the effect of muscle thixotropy on position-mirroring ability [22, 33, 34]. Following, the forearms were rotated to the central angle (90°). Throughout the remaining movements, audio cues (‘In’, ‘Out’, ‘Stop’) played aloud indicating the actions that the participant should make at their reference forearm and indicator forearm, respectively. Their forearm movements were restricted to <10°/s to ensure comparable interaction speeds and, in turn, torque responses from each device [14, 35]. The participant was instructed to rotate their reference forearm to the first randomized angle, followed by the second randomized angle. The inclusion of these randomized angles ensured that the participant could not mirror forearm positions based on timing cues. Next, the participant was instructed to rotate their reference forearm to the reference target position (extension: 102.5°, flexion: 77.5°) and to hold their arm still for 4 s. Once the target position was reached, the custom robotic device changed its control algorithm in order to hold the forearm at the target position. Finally, the participant rotated their opposite indicator forearm to mirror the position of their reference forearm. The participant stated ‘match’ when their forearms were perceived as being in an identical mirrored position. This marked the end of the trial.

### Data collection

The participant was scheduled for two testing sessions, with each reference arm tested on a separate day. The reference arm first tested was randomized across participants.

#### Preparation

To begin each session, the participant was seated in the Biodex chair with straps across their shoulder and torso to restrict trunk movements. The participant’s reference and indicator forearm were affixed to the custom robotic device and custom measurement device, respectively, using a fiberglass cast (Reykjavik, Capital Region, Iceland).

#### Active range-of-motion

To confirm that a limited range-of-motion did not impact the participant’s ability to mirror their forearm positions, we quantified at the paretic elbow their active range-of-motion. The participant was requested to extend and flex as far as possible so that we could measure the maximum and minimum angle to which the participant could reach. The participant was required to have an active range-of-motion of at least 102.5° of extension and 77.5° of flexion in order to be tested at the extension and flexion target positions, respectively. In our post hoc analyses, we determined, based on inspection of the data, that the position-mirroring errors may be impacted by an individual having a limited range-of-motion, rather than a position-mirroring deficit, if the target could not be overshot by at least 10°. In turn, we did not include data for a participant at a reference target location if their active range-of-motion did not permit the target to be overshot by at least 10°. We mitigated the extent to which the participant with stroke was constrained in rotating their forearm(s) by supporting their arm weight throughout the experiment [36]. As such, the participant did not experience the abnormal flexion coupling that can occur about their paretic elbow when abducting about their paretic shoulder, which limits their range-of-motion [36]. An additional benefit to supporting the weight of the arms was avoiding changes in position-mirroring errors that could be introduced during weight-bearing movements [37–39].

#### Position-mirroring assessment

We quantified how accurately and precisely the participant could mirror their forearm positions. To prevent visual and auditory cues from aiding their performance, the participant wore an eye mask and noise-canceling headphones. The participant performed four sets of ten position-mirroring trials for a total of 40 trials. Each set of ten trials was comprised of a single mode of limb control (i.e., passive or active). Presentation order of the mode of limb control across the four sets was randomized to be either active-passive-passive-active or passive-active-active-passive. The first two trials of each set were practice. Trials three through ten were testing and included in the data analyses. Within each set of testing trials, four were assessing position-mirroring ability at the extension target position and four at the flexion target position. Presentation order of the target position was randomized within each set. A minimum one-minute break was included after the participant completed each set.

## Data analyses

### Data preparation

We quantified how accurately and precisely participants could mirror their forearms for each testing scenario. To begin, the error when mirroring forearm positions was calculated for every trial. This error was defined as the difference between the angle of the indicator forearm and reference forearm when the participant notified the experimenter that the position of their forearms was mirrored. A positive error indicated that the participant overshot the reference target position, and a negative error indicated that the participant undershot. To address whether learning, fatigue, and boredom were affecting the results, we visually inspected the errors as a function of trial to confirm that trends of increasing or decreasing errors were not evident across the eight testing trials.

Accuracy across the eight testing trials for each testing scenario was then evaluated using constant error and absolute error. Constant error (CE) was the mean error across the eight testing trials within a testing scenario. A CE value greater than zero indicated that the participant, on average, overshot, less than zero that the participant, on average, undershot, and equal to zero that the participant, on average, perfectly mirrored the target position. Absolute error (AE) was the mean magnitude of error across the eight testing trials within a testing scenario. A large AE indicated that the participant, on average, mirrored their forearms with a substantial amount of error, and an AE equal to zero indicated that the participant always perfectly mirrored the target position.

Precision across the eight testing trials for each testing scenario was evaluated using variable error (VE) [14, 40, 41]. VE was the standard deviation of the error across the eight testing trials within a testing scenario. VE > >0 indicated that the position at which the participant mirrored was highly inconsistent, and VE = 0 indicated that the position was always exactly the same.

### Classification of a position-mirroring deficit

We classified whether each participant had a deficit in mirroring their forearm positions during the passive movements and active movements. Normative thresholds were obtained by applying the analytical methods outlined in [8] to the data from the similarly-aged individuals without neurological impairments (i.e., controls) who were assessed on the same conditions [14]. The methods are as follows. For each mode of limb control, the mean and standard deviation of the AE across both reference arms and target positions was calculated from the controls’ data [14]. Then, the passive and active threshold error was defined as their mean AE plus three standard deviations during the passive and active testing scenarios, respectively. A participant with stroke was classified as having a passive or active position-mirroring deficit if their AE exceeded the passive or active threshold error, respectively, when referencing either arm at either target position. Otherwise, the participant was classified as having intact passive or active position-mirroring ability.

### Error depending on the arm referenced

A goal of our analyses was to determine the extent to which the arm referenced influenced how accurately and precisely participants could mirror their forearm positions. Running statistical analyses using CE, AE, and VE could lead to misleading results since these outcome measures are averaged for each arm that participants reference, separately, prior to identifying whether the arm referenced influenced the findings. Hence, the grouped analyses may obscure individual differences in errors for when the non-paretic arm versus paretic arm is referenced. Therefore, we ran our analyses on outcome measures that quantify the extent to which each participant’s position-mirroring accuracy and precision changed depending on the arm referenced. Specifically, the three outcome measures we used were CE_diff_, AE_diff_, and VE_diff_.

The difference in constant error, CE_diff_ = CE_non−paretic_ − CE_paretic_, indicated for each condition whether the extent to which participants with stroke overshot or undershot when mirroring their forearm positions was influenced by the arm referenced. A value of CE_diff_ that was greater than zero indicated that the extent to which participants with stroke, on average, overshot when mirroring forearm positions was greater when referencing their non-paretic arm than their paretic arm. A value of CE_diff_ that was less than zero indicated that the extent to which participants with stroke, on average, overshot when mirroring forearm positions was greater when referencing their paretic arm than their non-paretic arm. A value of CE_diff_ that was equal to zero indicated that participants with stroke, on average, overshot or undershot when mirroring forearm positions to the same extent when referencing their non-paretic arm and paretic arm. For the data of the controls, the difference was defined as CE_diff_ = CE_dominant_ − CE_non−dominant_, with CE_dominant_ replacing CE_non−paretic_ and CE_non−dominant_ replacing CE_paretic_.

The difference in absolute error, AE_diff_ = AE_non−paretic_ − AE_paretic_, indicated for each condition whether the magnitude of errors when participants with stroke mirrored their forearm positions was impacted by the arm referenced. A value of AE_diff_ that was positive indicated that the magnitude of errors were, on average, greater when participants with stroke referenced their non-paretic arm rather than their paretic arm to mirror forearm positions. A value of AE_diff_ that was negative indicated that the magnitude of errors was, on average, greater when participants with stroke referenced their paretic arm rather than their non-paretic arm to mirror forearm positions. A value of AE_diff_ that was zero indicated that the magnitude of errors was, on average, the same when participants with stroke referenced their non-paretic arm and their paretic arm to mirror forearm positions. For the data of the controls, the difference was defined as AE_diff_ = AE_dominant_ − AE_non−dominant_, with AE_dominant_ replacing AE_non−paretic_ and AE_non−dominant_ replacing AE_paretic_.

The difference in variable error, VE_diff_ = VE_non−paretic_ − VE_paretic_, indicates the extent to which the variability in errors was impacted by the arm that participants with stroke referenced when mirroring forearm positions. A value of VE_diff_ that was positive indicated that participants with stroke, on average, mirrored forearm positions with more variability when referencing their non-paretic arm rather than their paretic arm. A value of VE_diff_ that was negative indicated that participants with stroke, on average, mirrored forearm positions with greater variability when referencing their paretic arm rather than their non-paretic arm. A value of VE_diff_ that was equal to zero indicated that participants with stroke, on average, mirrored forearm positions with the same amount of variability when referencing their non-paretic arm and their paretic arm. For the data of the controls, the difference was defined as VE_diff_ = VE_dominant_ − VE_non−dominant_, with VE_dominant_ replacing VE_non−paretic_ and VE_non−dominant_ replacing VE_paretic_.

### Statistical analyses

We determined to what extent the arm referenced influenced how accurately and precisely participants could mirror their forearm positions at each reference target position during the passive and active movements. This was achieved by determining whether the outcome measures of CE_diff_, AE_diff_, and VE_diff_ significantly differed depending on the testing condition. The analysis was run in the language R using the ‘Linear and Nonlinear Mixed Effects Models’ package *nlme*, version 3.1-128 [42, 43]. Data were fit to a linear mixed-effects model using the function *nlme*, with group classification (controls/participants with stroke: intact/participants with stroke: deficit), mode of limb control (active/passive), and reference target position (extension/flexion) as fixed effects and participant as a random effect. Following, an analysis of variance was run using the function *anova.lme* to identify significant main effect(s). Normality was checked by visually inspecting the linearity of the residuals on a normal quantile-quantile plot.

## Results

### Participants

Data were collected from the same eighteen participants with hemiparetic stroke who participated in our previous study [8]. Information about each participant is summarized in Table 1. The mean (standard deviation; range) of participant age was 60 (9; 42-75), and years post stroke was 12 (8; 3-29). Six participants were female and twelve were male, thirteen were right-hand dominant and five left-hand dominant, and nine had a paretic right arm and nine a paretic left arm. Participants were identified as having mild to severe motor impairments, with a mean (standard deviation; range) UE FMA score of 29 (14; 11-59).

**Table 1:** Participant information. Provided for each participant is their demographic and clinical information, as well as our classification for their position-mirroring ability during the passive and active movements.

Participant	Position-Mirroring Classification (Passive/Active)	Gender	Age	Dominant/Paretic Arm	Years since Stroke	Upper-Extremity FMA Score	Lesion Location(s)
Stroke 1	(Intact/Intact)	F	67	R/R	12	11	L: Th, IC, BG
Stroke 2	(Intact/Deficit)	M	61	L/R	12	50	L: IC
Stroke 3	(Intact/Intact)	F	61	R/L	3	28	R: IC, BG
Stroke 4	(Intact/Intact)	M	71	L/R	5	32	L: Po
Stroke 5	(Intact/Intact)	M	42	R/R	3	39	L: BG, T, F, P
Stroke 6	(Intact/Deficit)	F	69	R/L	16	28	NA
Stroke 7	(Deficit/Deficit)	M	63	R/R	17	59	NA
Stroke 8	(Intact/Intact)	M	61	L/L	6	17	R: IC, Po
Stroke 9	(Deficit/Intact)	M	46	R/L	11	35	R: Th, IC
Stroke 10	(Intact/Intact)	M	69	L/R	21	12	L: Th, IC, BG, I
Stroke 11	(Intact/Deficit)	F	75	R/R	12	56	L: F
Stroke 12	(Deficit/Deficit)	F	46	R/L	9	28	R: IC, SF, FP, T
Stroke 13	(Deficit/Deficit)	M	50	R/L	17	30	R: Th, IC, BG, PO
Stroke 14	(Deficit/Deficit)	M	60	R/L	4	20	R: BG, F
Stroke 15	(Intact/Intact)	M	59	R/L	9	25	R: Th, IC, BG
Stroke 16	(Deficit/Deficit)	M	49	L/R	27	26	NA
Stroke 17	(Intact/Intact)	F	62	R/R	29	13	L: Th, IC, BG
Stroke 18	(Intact/Intact)	M	61	R/L	8	15	R: IC, BG

FMA: Fügl-Meyer Motor Assessment, Min: minimum, Max: maximum, NA: information not available, Th: thalamus, IC: internal capsule, BG: basal ganglia, F: frontal lobe, FP: frontal/parietal lobes, PO: parietal/occipital lobes; I: insula, T: temporal lobe, P: parietal lobe, SF: sylvian fissure, Po: pons, EC: external capsule, CR: coronoradiata.

### Participant classification

We classified whether each participant had a deficit in mirroring the position of their forearms using the approach described in the ‘Data Analyses’ section. Six of the eighteen participants were classified as having a deficit during the passive movements and eight during the active movements. Stroke 2, 6, and 11 were identified as having intact perception during the passive movements, yet a deficit during the active movements. Stroke 9 was identified as having a deficit during the passive movements, yet intact perception during the active movements. The remainder of the participants were classified as having either only a deficit or only intact perception for both modes of limb control.

The passive threshold error was 8.2°. Hence, participants with stroke who had an absolute error greater than this passive threshold error during one of the four testing scenarios, i.e. referencing either arm at the extension or flexion reference target position, were classified as having a passive position-mirroring deficit. Table 2 summarizes the testing scenarios which resulted in the six participants being classified as having a deficit. We point out that the number of testing scenarios which can contribute to participants being classified as having a deficit is greater than one (e.g., both flexion and extension target positions); hence, the number of testing scenarios indicated in the table exceeds the number of participants classified as having a deficit.

**Table 2:** Testing scenarios for which participants were classified as having a deficit. Summarized are the number of incidences when referencing each arm at each target position for which participants were classified as having a deficit when their forearms were passively rotated and actively controlled.

Target Position	Passive		Active	
	Extension	Flexion	Extension	Flexion
Arm Referenced				
Paretic	3	4	6	6
Non-Paretic	4	1	6	3

The active threshold error was 11.4°. Hence, participants with stroke who had an absolute error greater than this active threshold error during one of the four testing scenarios were classified as having an active position-mirroring deficit. Table 2 summarizes the testing scenarios which resulted in the eight participants being classified as having a deficit.

### Errors in mirroring positions

Stroke 18 was not tested at the extension target position due to an inability to reach this position. Additionally, we did not include the results of Stroke 15 in extension and Stroke 10 in flexion since these participants could not overshoot the respective target positions by 10° during active limb control. As such, sixteen of the eighteen participants were assessed in extension and seventeen in flexion. Additionally, three trials were removed because of an experimental error during the active mode of limb control. Therefore, the following analyses were run on the remaining 816 and 813 trials for the passive and active movements, respectively.

#### Accuracy and precision in mirroring forearm positions

We provide an overview of how accurately and precisely our participants with hemiparetic stroke could mirror their forearm positions in Table 3. This table summarizes the errors when participants’ forearms were moved passively and actively to each reference target position when referencing each arm.

**Table 3:** Summarized errors. Reported for each classified group of participants when referencing each arm during passive and active movements are the mean (standard deviation) of the constant error, absolute error, and variable error.

Direction	Group	Arm Referenced	Passive			Active		
			CE [°]	AE [°]	VE [°]	CE [°]	AE [°]	VE [°]
Extension	Participants with	Non-Paretic	2.0 (3.7)	3.9 (1.8)	3.0 (0.9)	1.2 (3.7)	3.6 (2.5)	3.2 (1.5)
	Stroke: Intact	Paretic	1.6 (3.3)	3.4 (1.7)	2.8 (1.3)	3.4 (3.2)	5.1 (2.5)	4.4 (2.4)
	Participants with	Non-Paretic	7.6 (5.2)	8.9 (5.0)	6.1 (4.9)	8.5 (3.0)	8.8 (2.8)	4.8 (1.6)
	Stroke: Deficit	Paretic	5.7 (8.2)	9.5 (5.5)	7.3 (3.3)	9.8 (5.4)	10.4 (4.7)	5.6 (3.2)
Flexion	Participants with	Non-Paretic	1.0 (3.9)	3.6 (2.0)	2.5 (0.7)	2.1 (4.7)	4.1 (3.2)	2.0 (0.7)
	Stroke: Intact	Paretic	0.9 (3.2)	3.7 (1.2)	3.2 (1.5)	5.7 (4.1)	6.6 (2.5)	3.4 (1.1)
	Participants with	Non-Paretic	-1.6 (7.8)	6.2 (4.6)	4.5 (1.9)	0.2 (5.7)	5.4 (3.1)	4.0 (1.4)
	Stroke: Deficit	Paretic	2.8 (11.9)	10.0 (6.5)	5.3 (4.1)	8.7 (5.6)	9.4 (4.2)	3.4 (1.1)

#### Quantification of extent to which the arm referenced affects the outcome measures

**Fig 2:**
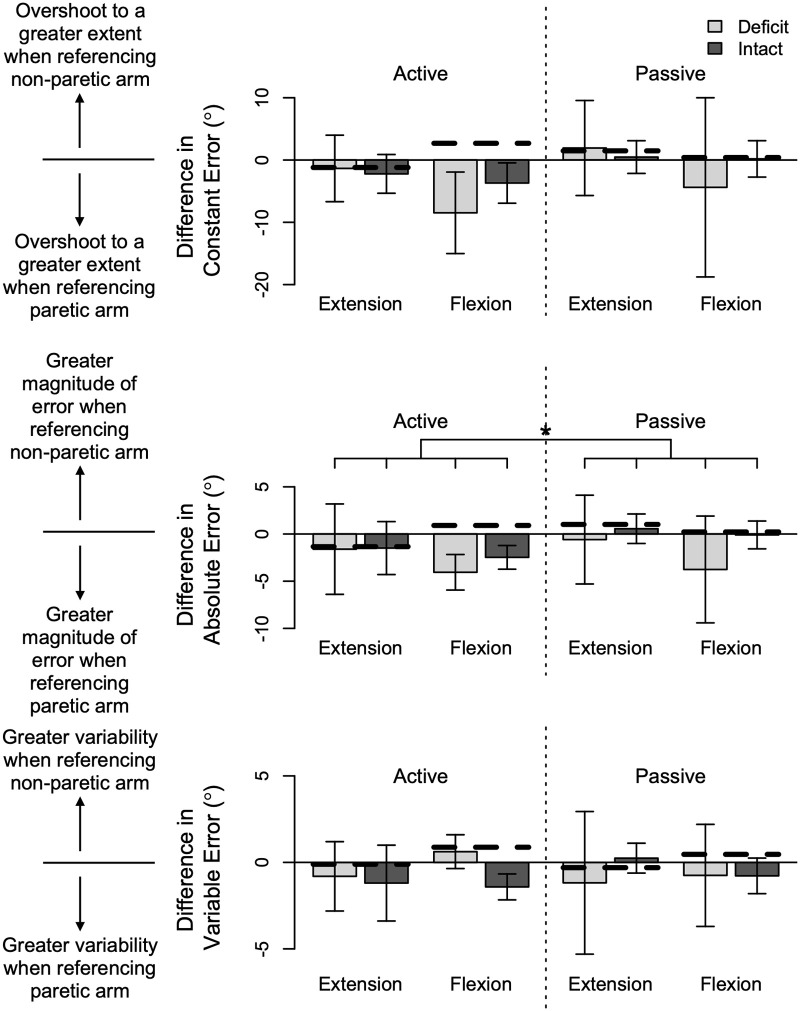
Summarized results. Reported for every testing condition of our participants are the mean (bar height) and 95% confidence interval (error bars) of the difference when referencing the non-paretic versus paretic arm in constant error, absolute error, and variable error. We also identify with the bold, dashed black lines the mean difference when referencing the dominant versus non-dominant arm for every testing condition of the controls in © Gurari et al. 2018 [14]. A significant difference between the active and passive movements of our participants with stroke and the previously tested controls is indicated by a line with a star above (p = 0.016).

We quantified the extent to which the arm referenced influenced how accurately and precisely our participants with hemiparetic stroke could mirror their forearm positions during the four testing conditions. Results summarizing the performance of our participants with stroke in relation to the controls are summarized in Fig 2.

The difference in the extent to which the target position was overshot when referencing the dominant/non-paretic arm versus non-dominant/paretic arm (CE_diff_) was not found to change depending on the group classification (i.e., controls/participants with stroke: intact/participants with stroke: deficit) (F(2,71) = 2.463; p = 0.092). That is, the classification as having a deficit or being intact, versus being a control, was not found to correspond to whether participants overshot/undershot to a greater extent when referencing their dominant/non-paretic versus non-dominant/paretic arm. Analyses also revealed that CE_diff_ was not significantly affected by the mode of limb control (active/passive) (F(1,71) = 2.903; p = 0.093) and reference target position (extension/flexion) (F(1,71) = 1.589; p = 0.212). Hence, the difference in errors depending on the arm referenced was not found to be influenced by participants actively controlling their movements versus having their forearms moved for them at an extended or flexed position.

Likewise, the difference in the magnitude of the errors when referencing the dominant/non-paretic versus non-dominant/paretic arm (AE_diff_) was not found to be influenced by the group classification (controls/participants with stroke: intact/participants with stroke: deficit) (F(2,71) = 1.325; p = 0.272). Moreover, AE_diff_ was not found to be significantly affected by the reference target position (extension/flexion) (F(1,71) = 3.086; p = 0.083). These results indicate that the difference in the magnitude of the errors when referencing the dominant/non-paretic versus non-dominant/paretic arm was not associated with whether the participant was classified as having intact perception or a deficit, or being a control, and whether their forearm was at the extension or flexion target position. In contrast, AE_diff_ was significantly affected by the mode of limb control (F(1,71) = 6.045; p = 0.016); the mean (standard deviation) of AE_diff_ was a greater magnitude and more negative during active limb control (-1.6° (3.7°)) than passive limb control (-0.2° (3.7°)). This significant finding indicates that the extent to which the magnitude of the errors was larger when referencing the non-dominant/paretic arm than the dominant/non-paretic arm was greater during active movements than passive movements.

The difference in the variability when referencing the dominant/non-paretic versus non-dominant/paretic arm (VE_diff_) to mirror forearm positions did not significantly differ depending on the group classification (controls/participants with stroke: intact/participants with stroke: deficit) (F(2,71) = 1.523; p = 0.225). That is, the classification of having a deficit or being intact, or being a control, was not found to correspond to whether participants mirrored forearm positions more or less consistently when referencing their dominant/non-paretic arm in comparison to their non-dominant/paretic arm. In addition, the difference in the mirroring variability when referencing the dominant/non-paretic versus non-dominant/paretic arm was not found to be significantly impacted by the mode of limb control (active/passive) (F(1,71) = 0.012; p = 0.912) and reference target position (extension/flexion) (F(1,71) = 0.769; p = 0.384). These results indicate that the difference in the variability of the errors when referencing the dominant/non-paretic versus non-dominant/paretic arm did not correspond to whether the participant’s forearm was actively controlled or passively rotated, and whether their forearm was at the extension or flexion target position.

## Discussion

This work is motivated by our desire to understand the impact of changing how a task is performed on whether an individual with stroke is classified as having a deficit in mirroring forearm positions. Our previous work demonstrated that individuals post-hemiparetic stroke who have a deficit when mirroring their forearm positions on a between-arms task may accurately reproduce the position of their paretic forearm on a single-arm task [7, 8]. Moreover, Hirayama et al. showed that individuals with various types of unilateral lesions could locate their thumb when using their paretic limb to reach for their non-paretic thumb, yet not vice versa [24]. As such, we investigated whether this difference in position-mirroring errors, depending on the arm referenced, extended to individuals with hemiparetic stroke. In particular, we were interested in assessing perception during active movements as previous studies have focused on perception of individuals with stroke when the paretic arm is passively moved. Given that activities of daily living often require active control of one’s limbs, a primary goal of this work was to assess perception in individuals with hemiparetic stroke when they controlled their own movements.

### Classification of deficits

We underscore that motor impairments in the tested participants did not impact our results. Our analyses only included data from participants with stroke who had an active range-of-motion in their paretic elbow that was sufficient to overshoot the position of their non-paretic forearm by at least 10°. Furthermore, range-of-motion limitations that occur in individuals post-hemiparetic stroke when extending their paretic elbow while abducting at their paretic shoulder were avoided by supporting the weight of the participant’s paretic arm [36].

The threshold error used to classify participants with stroke as having a deficit in mirroring forearm positions slightly differed for the passive (∼8°) and active (∼11°) movements. Even so, these angles are similar to the ∼10° threshold error that was identified on a task comprised of a passive movement at the reference forearm followed by an active movement at the indicator forearm (see [8]), as well as the 10° threshold error used in the revised Nottingham Sensory Assessment kinaesthesia test [4].

Our results also suggest that there may be a hierarchy of perceptual severity; the accuracy of individuals with stroke in judging their arm positions may be more severely affected if a deficit is found during passive movements than if found during active movements [44]. Eight of the eighteen participants with stroke were classified as having a position-mirroring deficit during the active movements whereas only six during the passive movements, despite the lower threshold error in the latter. Five participants who were classified as having a deficit during passive movements were also classified as having a deficit during active movements. Moreover, three of the participants who were classified as having intact perception during the passive movements were classified as having a deficit during active movements. Even so, one of the participants classified as having a deficit during the active movements was classified as having intact perception during the passive movements. That is, a deficit during active movements did not necessarily indicate a deficit during passive movements, and vice versa. Therefore, these findings indicate the importance of assessing participants under solely passive and solely active movements to obtain a more complete understanding of the extent of their deficits during different scenarios.

The number of times for which participants were classified as having a deficit based on each scenario was similar when participants referenced their non-paretic arm and paretic arm in extension, and paretic arm in flexion (see Table 2). Interestingly, the number of times for which an individual was classified as having a deficit when referencing their non-paretic arm in flexion was less than these other scenarios. The average constant error at the flexed position when referencing the non-paretic arm was -1.6° and 0.2° during passive and active movements, respectively. Our earlier results in controls when referencing their dominant arm at the flexed position have similar errors to the participants with stroke classified a having a deficit at the flexed position. The average constant error across all controls at the flexed position was 0.4° during passive movements and -0.1° during active movements [14]. Future work can probe further at whether individuals with stroke can accurately mirror forearm positions at a flexed position when referencing their non-paretic forearm during active movements. This knowledge of when a deficit occurs, and doesn’t occur, could be useful when considering how to provide rehabilitative therapies to individuals post stroke.

### Position-mirroring results

Our findings are in contrast to the findings of Hirayama et al. from their testing in individuals with various types of unilateral lesions [24]. Our results do not reveal a strong dependency on the arm referenced in how accurately individuals with hemiparetic stroke mirrored the position of their forearms during a bimanual task. The difference in the magnitude of the errors when controls and participants with stroke referenced their dominant/non-paretic arm versus non-dominant/paretic arm was affected by the mode of limb control, i.e., passive versus active (p = 0.016). However, the magnitude of difference was relatively small, with participants, on average, overshooting when referencing their non-dominant/paretic arm in comparison to their dominant/non-paretic arm by 1.6° during the active movements and 0.2° during the passive movements. Moreover, the difference in errors depending on the arm referenced was not found to change if participants were mirroring their forearm at the extension position (102.5°) or flexion position (77.5°) (p>0.050).

The lack of significance may be due to the fact that analyses were run after grouping data i) at the flexion and extension positions ii) during the passive and active conditions iii) with participants with stroke classified as having a deficit and being intact, as well as controls. Hence, the sample size of eighteen participants with stroke may not have been large enough to identify a significant difference. The findings at the flexed position during active movements reveal a mean (standard deviation) constant error when referencing the non-paretic arm of 0.2° (5.7°) and paretic arm of 8.7° (5.6°). Therefore, while significance was not found, this result supports the notion that accuracy in mirroring positions could differ depending on the arm referenced for this testing scenario. That is, individuals classified as having a deficit, on average, accurately mirrored forearm positions during active movements to the flexed position when referencing their non-paretic arm, yet inaccurately mirrored forearm positions when referencing their paretic arm. Thus, data collected from a larger sample size and/or testing this scenario alone may reveal a dependency on the arm referenced in one’s accuracy when mirroring forearm positions post stroke.

### Potential reason(s) for deficits

While we can only speculate, we will provide a brief discussion about the potential mechanism(s) contributing to the significant differences in results during the active and passive modes of limb control.

To begin, we highlight that motor deficits, as evaluated using the Fügl-Meyer motor assessment score, appear not to be related with the position-mirroring deficits we found in our sample. As indicated in Table 1, individuals who had severe motor deficits were classified as having intact perception, and vice versa. For example, Stroke 1 and Stroke 8 had severe motor deficits yet were classified as being intact when mirroring forearm positions. Contrastingly, Stroke 7 and Stroke 11 had mild motor deficits and were classified as having deficits in mirroring forearm positions.

Additionally, we do not have a reason to believe that peripheral inputs contributed to the significant result between the active and passive modes of limb control. The slow movement speeds of 6°/s (passive) and less then 10°/s (active) were selected in part since they are slow enough to avoid evoking hyperactive stretch reflex activity, which is known to exist in individuals post stroke [7, 45]. Hence, there is no reason to believe that peripheral inputs differed when participants controlled their forearms versus a device rotated their forearms, regardless of the arm referenced.

Another consideration is that active movements of one forearm may result in involuntary movements of the contralateral forearm, termed mirror movements, which may have affected perception [46]. We did not consider mirror movements in our protocol and analyses. As such, we fixed the non-rotating arm in our protocol such that only one arm was permitted to rotate at a time. Moreover, we did not investigate whether forces and muscle activity were generated at the arm that was fixed in place. Hence, we cannot address whether mirror movements occurred and, if yes, were perceived. It is important to consider, however, that greater differences in errors when mirroring forearm positions, during active versus passive movements, were also identified in similarly-aged controls who do not generate mirror movements. Future work can address whether these mirrored movements differentially affect how accurately forearm positions can be mirrored depending on the arm referenced.

We provide several explanations to address why the significant result, depending on whether movements were active or passive, was obtained. First, during passive movements, feed-forward sensory-related information (e.g., efference copy) is absent since an individual is not actively controlling their limb(s). During active movements, our participants could rely on both the sensory and feed-forward sensory-related information to mirror their forearm positions [19, 29–31]. As such, the feed-forward sensory-related information may have been inaccurate and contributed to greater differences depending on the arm referenced during the active movements than the passive movements (see Table 3). Second, the cognitive load may have been greater during the active versus passive movements, and differentially impacted as a function of the arm referenced. Thus, the cognitive load may have contributed to the greater differences in errors during the active as opposed to passive movements.

Regarding the neural mechanisms underlying the position-mirroring deficits that are seen in nine of the eighteen participants with stroke, these may be related to sensorimotor and cognitive neural structures affected by the stroke. Nearly all of the participants classified as having a deficit had lesions in the internal capsule, thalamus, and/or basal ganglia. However, Stroke 11, who was classified as having a deficit during active movements, had a lesion in the frontal lobe. Moreover, participants who were classified as not having a deficit when mirroring forearm positions also had lesions in the internal capsule, thalamus, and basal ganglia. Therefore, our results, based on a limited sample size, do not elucidate specific neural substrates. Furthermore, our knowledge about the brain damage is limited to the affected anatomical structures as determined based on visual inspection of anatomical MR images. Future work can use more precise and comprehensive neuroimaging techniques, such as high-resolution diffusion weighted structural MRI [47–49], to report on the damage that has occurred to the brain. In turn, an understanding may be obtained that extends beyond lesion location(s) in the brain, based on visual inspection of anatomical MR, to changes in structural integrity of neural pathways that can potentially affect function.

Moving forward, we reiterate that outcomes on an assessment can change depending on how a task is performed, e.g., single arm versus between arms [7, 8], passive versus active movements. Therefore, identifying neural substrates that generalize to differing tasks may prove challenging. Future work can link neural substrates to deficits in perceiving where one’s limb is positioned based on outcomes from various tasks, that include purely active and purely passive movements. In turn, the identified neural substrates that may contribute to limb position perceptual deficits can be generalized across a range of activities.

### Limitations

One of the limitations in this area of research is the poor understanding for how to appropriately classify a proprioceptive deficit, e.g., [5, 50]. This point is underscored by our earlier work in which we discovered that a deficit in mirroring forearm positions on a between-arms task does not necessarily indicate that an individual with stroke will have a deficit when matching forearm positions using their paretic forearm [7, 8]. Furthermore, as indicated in this work, classification of a deficit can differ depending on whether movements are solely passive versus solely active. Moreover, we acknowledge that individuals may be classified differently depending on the outcome measure, such as accuracy (e.g., absolute error) versus precision (e.g., variable error). Aligned with the goals of other studies, we aimed to classify participants as having a deficit based on our quantitative measures [3, 27]. As such, we classified our participants as having a deficit in their position-mirroring ability by using our previously-defined approach [8]. Once future work determines the reason why a deficit arises, we will be better equipped to define targeted assessment(s) and appropriate outcome measure(s) to characterize proprioception in individuals with hemiparetic stroke.

We also point out that participant recruitment was restricted to individuals with stroke who could detect the direction in which their forearm was passively rotated. This group of participants, hence, differs from the example individual shown in our supporting video (S1 Video). To successfully complete the passive mode of limb control portion of our study, participants needed to be able to detect the direction of their paretic forearm’s movements when passively imposed. Therefore, by restricting our participant recruitment to individuals with hemiparetic stroke who could detect the direction of rotation at their paretic forearm during passive movements, we could assess position-mirroring ability during solely passive movements in addition to during solely active movements. Even so, we acknowledge that by including this passive mode of limb control in our study design, we excluded individuals who present with more severe deficits during passive testing. Future work can assess individuals with stroke who are unable to detect the direction of rotation at their paretic forearm during passive movements on the active protocol. Findings will indicate whether the extent to which the arm referenced impacts accuracy is more pronounced in a population who presents with more severe passive perceptual deficits than the population tested here.

An additional limitation is that the results presented here are based on a heterogenous group of participants with stroke in terms of arm dominance and lesioned hemisphere. While the number of participants tested is relatively low, our analyses did not reveal a trend towards a significant effect of arm dominance and lesioned hemisphere on their accuracy and precision in mirroring forearm positions.

Moreover, another consideration is that cognitive deficits could be a potential confounding factor [51, 52]. All of our participants could understand and follow directions, as well as perform the position-mirroring task. Yet, we did not include formal outcome measures to more comprehensively characterize cognition. Hence, future work can include more defined inclusion/exclusion criteria and outcome measures relevant to cognitive function.

Finally, findings from our assessments may not extend to functional activities when the weight of the forearm is no longer supported. An individual typically abducts at their shoulder to perform simple tasks such as reaching for a cup or an object on a shelf. After a hemiparetic stroke, individuals with stroke may experience an unintended flexion torque at their paretic elbow, wrist, and fingers when abducting at their paretic shoulder, referred to as the flexion synergy [53, 54]. Future work can investigate how position-mirroring performance may be affected by the unintended elbow, wrist, and finger torques that occur when individuals post stroke abduct at their shoulder [36].

## Concluding remarks

This study provides further evidence that, post stroke, one’s classification on an assessment of *position sense* can depend on how an individual performs the task. For example, whether a movement is actively controlled or passively imposed, the non-paretic or paretic arm is referenced, and the arm is at a flexed or extended position can influence the classification. Future work can extend our active protocol to individuals with stroke who are unable to detect passively-imposed movements at their paretic limb. As such, we will have an improved understanding of the extent to which how a task performed during a position-mirroring assessment impacts whether an individual post stroke is classified as having a deficit.

## Supporting information

S1 Video Supplemental video demonstrating that the arm referenced can influence whether an individual with hemiparetic stroke can mirror forearm positions. The individual post-hemiparetic stroke shown in this video was classified as having absent kinaesthesia according to the revised Nottingham Sensory Assessment (rNSA) [4]. The rNSA task requires the individual to use their non-paretic forearm to mirror the position of their passively-placed paretic forearm. This individual could neither detect the position, nor movement at their paretic forearm; hence, this individual did not rotate their non-paretic forearm to mirror the position of their paretic forearm. In contrast, this individual could use their paretic forearm to roughly mirror the position of their passively-placed non-paretic forearm. The individual in this video has given written informed consent, as outlined in the PLOS consent form, to publish these case details.
(MP4)
